# The price of access: capitalization of neighborhood contextual factors

**DOI:** 10.1186/1479-5868-10-95

**Published:** 2013-08-08

**Authors:** Henry Shelton Brown, Lisa M Yarnell

**Affiliations:** 1 UTHealth School of Public Health, Austin Regional Campus Michael & Susan Dell Center for Healthy Living, Austin, TX, USA; 2 Department of Psychology, University of Southern California, Los Angeles, CA, USA

## Abstract

**Background:**

Studies of neighborhood context on health behavior have not considered that the health benefits of context may be ‘capitalized’ into, or included in, higher housing values. This study examines the associations of better neighborhood context with neighborhood housing values.

**Methods:**

We use the third wave of Add Health (2000-2001) to estimate the association of neighborhood contextual variables and housing values first across then within income types. This is a census block group-level analysis.

**Results:**

We find that neighborhood context, especially access to fruit and vegetable outlets, is capitalized into, or associated with, higher housing values. Fast food and convenience store access are associated with lower housing values. Capitalization differs by income quartile of the neighborhood. Even those in the poorest neighborhoods value access to fresh fruits and vegetables, and those in the wealthier neighborhoods value activity resources. All neighborhood incomes types place negative value on fast food access and convenience store access.

**Conclusions:**

Access to health-related contextual attributes is capitalized into higher housing prices. Access to fresh fruits and vegetables is valued in neighborhoods of all income levels. Modeling these associations by neighborhood income levels helps explain the mixed results in the literature on the built environment in terms of linking health outcomes to access.

## Background

Neighborhood context, defined as the physical and social attributes of a neighborhood, is correlated with health [1]. For instance, better relative access to healthy food and physical activity (PA) resources within a neighborhood is associated with lower body weight at the individual level. This is likely because better access lowers the time required to access healthy food and PA [2-6]. However in most neighborhood context studies, the association between health effects and context are estimated assuming that locational decisions by individuals are random, which leads to bias [7,8]. An important and helpful theory from urban economics has been ignored in this literature: the possibility that the health benefits of local access to healthy food/PA, and of better neighborhoods generally, may be capitalized into, or included in, higher housing values. Likewise, proximity to health-compromising features, such as fast food outlets, may be negatively capitalized, or discounted in terms of housing values or rent.

Capitalization can be thought of as evidence of preference, at the aggregate level, for a given neighborhood and its attributes as compared to alternative neighborhoods. Housing price models are bid models. For instance, if a neighborhood acquires an attribute, like a redeveloped waterfront, its increased desirability will encourage higher bids for the homes in that neighborhood, leading to higher costs for buyers to live there. With capitalization, ‘premiums,’ or the portion of the housing price which is attributable to that locational feature, may be required for access. Homes can also be ‘discounted’ (receive a negative premium) for features negatively related to health, indicating underlying preferences for health by those willing to pay for access.

It is acknowledged that income and health are highly correlated [1]. The paradox is that if access to “better” neighborhood contextual factors is capitalized, income, net of housing costs, will be reduced, which might lower health outcomes. Therefore, the overall effects of contextual factors on health is ambiguous. Further, if neighborhood contextual factors are shown to be capitalized, this demonstrates the extent to which locational decisions are not random. Neither issue has been given sufficient attention in the contextual factor literature.

Two economic theories suggest that housing markets capitalize the benefits of neighborhood features. First, von Thünen theorized in the early 19^*th*^ century that land values are a function of the local area’s attributes, particularly, access to the center of the city, rather than the intrinsic value of the property in isolation [9]. Second, the Tiebout model of the 1950’s asserted that people sort themselves among localities in order to find their utility-maximizing mix of government services, many of which affect health, and local taxes [10]. Thus, people with similar tastes and similar norms, including ‘like-mindedness’ over health issues, are likely to sort together.

Empirically, ‘hedonic’ price models have been used to estimate the level of capitalization of local attributes and amenities. For example, if two identical houses on two identical lots in different areas of town are priced differently, differential local contextual factors, like schools or crime level, may explain the difference in price or capitalization. Hedonic models have been used to estimate the capitalization of better local schools [11], of relative crime avoidance [12], and of relative environmental advantages such as clean air [13]. However, studies of capitalization in the neighborhood context literature do not exist.

We address this gap in the literature by estimating the association of neighborhood contextual variables–particularly, health-related neighborhood contextual variables–and housing values, using the third wave of data from the National Longitudinal Study of Adolescent Health (Add Health), reflecting the years 2000-2001. In our hedonic regressions of housing values by income quartile, we demonstrate that health-related attributes such as supermarket density are capitalized.

### Neighborhood context and health

Health-related neighborhood attributes have received particular attention in sociological and economic literature as correlates of health, yet, as mentioned, have not been investigated explicitly in models of housing capitalization. For instance, access to healthy food has been linked to better diets and lower obesity rates. There is extensive literature documenting that poorer and ethnic minority neighborhoods have fewer supermarkets per household [14-23]. Supermarkets often provide higher quality food products and offer lower food prices than other food outlets [22,24]. Increased availability of chain supermarkets is significantly associated with lower adolescent BMI and overweight, while greater availability of convenience stores is significantly associated with higher adolescent BMI and overweight [23]. According to Powell et al. (2007), the link between supermarket availability and weight is often indirect, with studies often simply demonstrating that availability is associated with consumption [25], or that availability is associated with diet quality [26].

There is also extensive literature documenting that particular physical environments are positively associated with PA levels [4-6]. Neighborhoods with higher income and lower poverty have more of certain PA outlets, including parks and bike path lanes (though not more public pools or beaches) [27]. Lower income individuals are more likely to perceive limitations to PA in their neighborhoods, including safety, gangs, traffic, and affordability [27,28]. Regular PA, in turn, is associated with reduced obesity [27,29].

### The bid model

The von Thünen bid model can be illustrated as follows. Suppose everyone living in the urban space must travel downtown for work. Commute times vary by location of residence, and people place different values on time, and that value is correlated with wages. Suppose that a person values time at $10 an hour, and would save 20 hours per month commuting downtown for work by moving from his current location to location *X*. What would he bid for the move? He would bid up to $200 ($10 times 20 hours) more than his current location. This may be enough to displace the person currently at location *X*, who may place a lower value on time. Alternatively, the person at location *X* may match the higher bid. According to the bid model, people bid and sort until an equilibrium emerges, where no one is induced to outbid people at other locations. Note that not everyone must move for the equilibrium to emerge.

All else equal, land rents or prices tend to increase towards the center of the city because of lowered commute time costs.^1^ This equilibrium pattern of rental and land prices is called a rental gradient. Rental gradients can also shift up or down because of characteristics other than time needed to travel to a nearby location. These include local area crime, pollution, school quality, etc. For instance, if crime avoidance is valued by the potential mover in the previous paragraph, he would bid less than $200 if location *X* has higher crime than his current location. If crime is lower at location X, he would bid more than $200. Note that the process of valuation is subjective, such as with the perceived threat of crime. Note also that rents and prices for homes are highly correlated, spatially; there are few places with high housing rents and low housing prices.

Note that income limits the ability to bid, regardless of preference for a local attribute. For instance, low-income people may highly value education for their children, but not be able to afford to live in areas with the very best schools. However, qualities such as crime level and school quality are reflected by a range of levels, rather than in binary variables. Therefore, low-income families may be willing, and able, to pay more to live in a location with average schools rather than living in an area with worse schools.

In this paper, we apply von Thünen’s bid model by testing whether access to local food and PA amenities that are positively related to health are shown to be valued positively in terms of housing prices, i.e., whether they are associated with an upward shift in the gradient, and by how much; and whether amenities negatively related to health are valued negatively in terms of housing prices. Because poorer people are likely to be outbid for access to locations with the absolute best access to healthy food and PA amenities, we stratify our analysis by income level of the neighborhood.

## Methods

### Data

Our data are from the third wave of data from the Add Health data set, a longitudinal study based on a nationally representative sample of adolescents in the United States, initially drawn in the 1994-1995 school year. In our study, we utilize data from Wave III of Add Health, in which a majority (73.13%) of the original Wave I respondents were re-interviewed between August 2001 and April 2002, when these participants were between 18 and 26 years old [30]. We would have liked to have taken advantage of the full panel of Add Health data. However, our capitalization and neighborhood context measures were only available at Wave III.

We chose the Add Health data set for this analysis for several reasons. Most important, the neighborhood amenities in the Add Health data are counted with respect to a distance, either straight line or street network, from an individual’s residence, at one of four levels: block group, tract, county, or state. We chose the block group level (keeping only one observation, randomly chosen, per block group); and used the street network distance, based on an 8 kilometer search, for the counts. A block group is defined by the U.S. Bureau of the Census as a “subdivision of a census tract... [and] the smallest geographic unit for which the census bureau tabulates sample data. A block group consists of all the blocks within a census tract with the same beginning number” [31]. Block groups average about 1,000 inhabitants.

U.S. Census data also counts amenities such as supermarkets and fast food outlets at the block group level. However, it does not base its counts on street network distances from each individual’s residence, but rather presents counts in summative fashion for the block group as a whole, regardless of the location of individual’s residences within that block group. Hence, the Add Health data, while used here at the neighborhood level only, is inherently individual-focused. Measuring counts based on travelable streets ties in directly with the von Thünen bid model, reflecting the time and effort a resident would practically spend to reach the desired neighborhood amenity, such as a supermarket. In analyzing neighborhoods or block groups rather than persons, we specified school ID as the primary sampling unit, and specified region as the strata variable, as indicated in Add Health weighting instructions. We used Stata’s subpopulation weighting techniques when estimating models separately by income quartile.

We also found that the Add Health data have good representativeness in terms of sampling. Add Health’s contextual data sample included low-population, rural areas with as few as 1900 persons per county at Wave III. For comparison, the American Community Survey (a yearly counterpart to the decennial U.S. Census) is less representative in terms of places represented in the data, being based on a more restrictive standard of 20,000 or more persons in a given area [31]. Additionally, the Add Health data contain health outcome variables that, in a next stage of this research, can be easily linked with the home values and neighborhood amenities, to determine the influence of these on health.

Finally, we chose to analyze data from earlier in the 2000s because this reflected a period just before the housing bubble, and then the subsequent housing crash [32]. Either event could bias attempts to correlate attributes with housing values.

The use of the data has been approved by the Committee for the Protection of Human Subjects at the University of Texas Health Science Center.

### Measures

#### ***Density***

We based population density on a Wave III contextual data item marking persons per square kilometer at the block group level. This variable should be correlated with distance to the urban center, making it essential in determining housing value in the von Thünen sense [9].

#### ***Per capita income***

We based per capita income on a Wave III contextual data item marking per capita income at the block group level. In Tiebout models, similar people sort together [10], so we would expect wealthier people to pay a premium to live near other wealthy people. In addition to using this item as a predictor of median housing value and rent, we also used it to divide our sample into quartiles based on block group per capita income. This was done using the PROC RANK procedure in SAS. Dummy variables created from this procedure (named ‘first’ for the poorest, ‘second’, ‘third’ and ‘fourth’ for the wealthiest) were used as subpopulation markers for Stata’s subpopulation command, which allowed us to estimate models separately by income quartile group.

#### ***Median year built for housing units***

We based median year that housing units in each block group were built on a Wave III contextual data item ranging from 1939 (reflecting a build year of 1939 or earlier) to 1999.

#### ***Median home value***

We based median home value on a Wave III contextual data item marking median home value of owner-occupied housing units at the block group level, based on Census 2000 data. Medians were preferable because this value was most representative of each block group, without undue influence by markedly higher or lower home values for a small number of blocks in the group. Utilizing values for owner-occupied homes excluded homes for which the owner would not be using its nearby amenities, such as rental units, vacation homes, or seasonal residences.

In the original Add Health data, 95% of the home values ranged from $16,700 to $935,700. A small number of cases had values of less than $10,000 or greater than $1,000,000. We eliminated cases that fell into these lower and higher categories due to their extremity and the crude grouping of these cases in the Add Health data. This provided us with a representative range of values without excessive extremes, and with relatively precise measurement. Finally, we divided these home values by 1,000 to yield a variable with a scale that was reasonably matched with the predictors, allowing for interpretable beta weights.

#### ***Densities of food and activity resources***

We based Wave III densities of supermarkets, fast food chain outlets, cooperative natural/health food stores, convenience stores, fruit/vegetable markets, and activity outlets on Wave III neighborhood resource data items marking counts of these resources within eight-kilometer network search units. Activity outlets included instructional facilities, membership facilities, outdoor facilities, public fee facilities, parks, public facilities (such as beaches and pools), schools, YMCAs, and youth organizations (such as boy and girl scout organizations). These resources had been categorized by Add Health staff according to Dun and Bradstreet’s 2001 primary Standard Industrial Classification (SIC) codes and keyword searches within the company name and trade style fields. While the Dun & Bradstreet data have had their accuracy questioned, recent literature has shown that the predictive validity of the D&B food resource data is “good” and comparable to competitor data such as from InfoUSA and Department of Health and Environmental Control data [33,34].

To convert counts into densities, we divided the counts by Census tract population (a tract being a small, relatively homogeneous subdivision of a county, larger than a block group) [31]. To put the density values on a scale similar to other variables in our models, we then multiplied these values by 1,000.

#### ***Total crime***

We based total crime on a Wave III item giving an index of total crimes reported (as opposed to arrests made) per 100,000, at the county level.

#### ***Proportion college educated***

We based proportion of persons with a college education on a Wave III variable marking the proportion of persons at the block group level who are 25 years or older and have a bachelor’s degree or more. In Tiebout models, similar people sort together [10], so, as with income, we would expect college-educated people to pay a premium to live near similar people.

#### ***Region***

Because real estate varies by region, we included dummy variables (1=yes, 0=no) for whether the block group was in the West, the Midwest or the South, with the Northeast as the omitted variable.

#### ***Activity by crime interaction***

Research has shown that in high-crime areas, parents are more apprehensive about using parks and trails [35], and value access less because they are seen as hosting criminal activity [12]. Prior to modeling, we also created a Crime by Activity Resource Density interaction term, having centered the Total Crime and Activity Resource Density variables prior to cross-multiplication. The interaction term was included in all specifications of our model, along with the centered versions of the component terms.

### Analysis

Our initial sample consisted of 7,870 unique block groups. Prior to analysis, we performed natural log transformations on the population density, activity density, and five food outlet density variables in order to account for the highly skewed nature of these variables. After these transformations, all variables showed reasonable skew statistics.

Next, we checked our data for outliers on the variables to be used in our regression models. We applied a conservative 6.0 standard deviation cutoff for the removal of outliers from our sample. Removal of cases with absolute values of greater than 6.0 standard deviations from the mean on one of more predictors reduced the sample to *N* = 7,845, and removal of additional cases with extreme values for home prices reduced the sample to its final size of *N* = 7,817.

Our hedonic price model is of the form

(1) $$Y_{b}={\text{XH}}_{b}\beta_{b}+{\text{XN}}_{b}\beta +u_{b},$$

where *Y*_*b*_ is the housing value for census block *b*, *XH*_*b*_ are health-related attributes of that location, including access to food, etc., *XN*_*b*_ are non-health-related attributes of that location, including access to the center of the city (density) and age of the housing stock. Finally, *u*_*b*_ is the error term.

We estimated this model first across all block groups, then separately by income quartile. As noted, we included Median Income as a predictor in the model even when running models separately by income quartile. Our reasoning is that even among income quartiles, there is still significant variation on the Median Income variable. We also ran models both with and without the natural/health food and fruit/vegetable market density variables, reasoning that these variables could be collinear with the supermarket density variable.

## Results

Table 1 shows the means and proportions employed in our analysis.

**Table 1:** Weighted means and proportions

**Variable**	**Mean, Proportion**	**(Std. err.)**
Median housing value, block group level	135.961	(5.430)
Population density, block group level	2765.224	(313.526)
Proportion 25+ with a college degree, block group level	0.245	(0.009)
Per capita income, block group level	20091.347	(447.524)
Median year housing built, block group level	1967.772	(1.025)
Northeast (1=yes; 0=no)	0.126	(0.022)
West (1=yes; 0=no)	0.264	(0.036)
Midwest (1=yes; 0=no)	0.233	(0.037)
South (1=yes; 0=no)	0.377	(0.042)
Supermarket density	5.624	(0.413)
Fast food (chain) density	22.804	(1.427)
Natural/health food store density	3.217	(0.171)
Convenience store density	36.723	(2.554)
Fruit/vegetable market density	2.506	(0.194)
Activity resource density	152.578	(8.837)
Crimes reported per 100,000, county level	4930.496	(155.710)
Activity resource density by Crime interaction	45.661	(62.847)
*N*	6,882	

Note: Units for Median Housing Value were thousands of 2001 dollars. Units for Population Density were persons/*km*^2^. Units for Per Capita Income were 2001 dollars. Units for resource density variables were 8 km count/tract population X 1000.

Table 2 shows the results for the hedonic regression model for housing values estimated across all income quartiles. As expected, block group level income is correlated with housing price, as is percentage of adults aged 25 and older and with college education. This is in accordance with predictions of the Tiebout model, where wealthier people pay a premium to sort together.^2^ This would as be predicted if there is a premium for prestige in a neighborhood, or hard to observe benefits that accrue from living near wealthier, educated people. All else equal, housing costs more in the West and less in the South and Midwest. Also as expected, the crime rate is negatively associated with housing value, and block group level density is positively related to housing value. As mentioned earlier, population density is a proxy measure for distance to the center of the urban space in the von Thünen sense [9]. The greater the density, the greater the housing price because, typically, this is closer to the central city relative to less dense areas, thus affording greater access.

**Table 2:** Hedonic regressions predicting median home value, full sample of census blocks

**Variable**	**Coef**	**(Std. err.)**	**Coef**	**(Std. err.)**
Natural log of density, block group level	7.274^∗∗^	(1.490)	8.451^∗∗^	(1.729)
Proportion 25+ with a college degree, block group level	85.406^∗∗^	(11.347)	71.592^∗∗^	(11.981)
Per capita income	0.005^∗∗^	(0.000)	0.005^∗∗^	(0.000)
Median year housing built	0.020	(0.170)	-0.162	(0.173)
West (1=yes; 0=no)	37.030^∗^	(14.495)	30.147^*‡*^	(15.571)
Midwest (1=yes; 0=no)	- 16.261^*‡*^	(9.686)	- 25.836^∗^	(11.008)
South (1=yes; 0=no)	- 19.758^*‡*^	(10.401)	- 26.335^∗^	(11.230)
Natural log of supermarket density	-1.197	(2.529)	7.923^∗∗^	(2.959)
Natural log of natural/health food store density	2.463	(2.900)		
Natural log of fruit/vegetable market density	29.069^∗∗^	(3.428)		
Natural log of fast food (chain) density	- 6.251^∗∗^	(1.598)	- 8.274^∗∗^	(1.825)
Natural log of convenience store density	- 3.726^∗^	(1.825)	-0.629	(1.699)
Natural log of activity resource density, centered	1.797	(1.392)	4.414^∗∗^	(1.670)
Crimes reported per 100,000, county level, centered	- 0.004^∗∗^	(0.001)	- 0.005^∗∗^	(0.002)
Activity resource density by Crime interaction	0.000	(0.000)	0.000	(0.001)
Intercept	-68.104	(331.142)	291.836	(337.075)
*N*	6998		6998	
*R*^2^	0.615		0.589	
*F*	*F*_(15,114) _= 68.340		*F*_(13,116) _= 77.957	

Note: Numerator and denominator degrees of freedom for the *F* tests reflect, respectively, regression parameters minus 1 (*k*), and design degrees of freedom given the sampling frame, minus *k*. Significance levels: *‡* : 10% ∗ : 5% ∗∗ : 1%

In terms of health-related neighborhood contextual factors, most of the signs are as expected. Estimates for fruit/vegetable market density (positive), fast food density (negative), and convenience store density (negative) are significantly different from zero. Thus, neighborhood contextual factors are capitalized. Elasticities are useful in interpreting these coefficient estimates, particularly when both the dependent and independent variables are continuous. Elasticities measure the percentage change in the dependent variable associated with a percentage change in an independent variable. In the first model model, 10 percent rise in the density of fruit and vegetable stores is associated with $2,907 higher home values ($3884 in 2012 dollars). On the other hand, people pay a premium to the avoid convenience stores and fast food chains. A 10 percent rise in the density of fast food chains is associated with $625 lower home values ($824 in 2012 dollars).

Activity resource densities are not significantly related to housing values in the first model. As mentioned, research has shown that in high-crime areas, parks and trails are not valued because they are seen as host sites for criminal activity [12]. Therefore, we examined the interaction between crime rate and activity resource density. However, the interaction term was not significantly related to housing values in the first model.

In order to determine if supermarket density is positively associated with housing values when specialty food stores are not controlled for, the model on the right in Table 2 excludes natural food and fruit and vegetable store densities. Supermarket density is significantly associated with housing values when natural food and fruit and vegetable store densities are excluded. In this model, a 10 percent rise in the density of supermarkets is associated with $792 higher home values ($1,059 in 2012 dollars). Activity density is also significant when natural food and fruit and vegetable store densities are excluded. A 10 percent rise in activity densities is associated with $441 higher home values ($590 in 2012 dollars).

### Results by income quartile

Because tastes and preferences for access may differ by income, we also estimated our hedonic regression model separately by income quartile, with results shown in Table 3. Supermarket density is valued amongst block groups in the 1^*st*^ (lowest) quartile of income when natural food and fruit and vegetable store densities are excluded. A 10 percent increase in supermarket density is associated with $1,128 increased housing values in the 1^*st*^ quartile ($1,477 in 2012 dollars). People living in neighborhoods in the 1^*st*^ income quartile in terms of income also value access to fruits and vegetable outlets, paying a hefty premium to have access. A 10 percent increase in fruit and vegetable store density is associated with $2,572 in increased housing values ($3,436 in 2012 dollars).

**Table 3:** Hedonic regressions predicting median home value, by income quartile

**Hedonic regressions for 1**^***st ***^**and 2**^***nd ***^**income quartiles**								
	***1***^***st ***^**Quartile**				***2***^***nd ***^**Quartile**			
**Variable**	**Coef**	**(Std. err.)**	**Coef**	**(Std. err.)**	**Coef**	**(Std. err.)**	**Coef**	**(Std. err.)**
Natural log of density, block group level	7.558^∗∗^	(1.330)	9.279^∗∗^	(1.516)	7.239^∗∗^	(1.172)	8.144^∗∗^	(1.240)
Proportion 25+ with a college degree	95.678^∗∗^	(12.174)	84.024^∗∗^	(13.238)	81.177^∗∗^	(16.515)	79.084^∗∗^	(16.764)
Per capita income, block group level	0.003^∗∗^	(0.001)	0.003^∗∗^	(0.001)	0.002^*‡*^	(0.001)	0.002	(0.001)
Median year housing built	0.313^∗^	(0.140)	0.283^∗^	(0.142)	0.5590^∗∗^	(0.104)	0.462^∗∗^	(0.115)
West (1=yes; 0=no)	17.376	(11.788)	9.846	(14.623)	12.755	(11.732)	7.506	(15.737)
Midwest (1=yes; 0=no)	-15.118	(13.840)	-25.199	(16.108)	- 22.134^*‡*^	(12.506)	- 30.016^∗^	(14.680)
South (1=yes; 0=no)	- 22.867^∗^	(11.255)	- 29.218^∗^	(13.670)	- 26.126^∗^	(10.714)	- 31.667^∗^	(13.353)
Natural log of supermarket density	-2.092	(2.796)	11.277^∗∗^	(2.492)	-3.629	(3.728)	5.812	(3.707)
Natural log of natural/health food store density	3.384	(3.421)			7.574^∗^	(3.731)		
Natural log of fruit/vegetable market density	25.715^∗∗^	(4.653)			21.378^∗∗^	(4.595)		
Natural log of fast food (chain) density	- 3.613^∗^	(1.783)	- 6.247^∗∗^	(1.988)	- 3.584^∗^	(1.447)	- 5.139^∗∗^	(1.539)
Natural log of convenience store density	-2.189	(1.651)	-2.112	(1.565)	-1.215	(1.819)	1.292	(1.820)
Natural log of activity resource density, centered	-1.250	(1.241)	0.739	(1.384)	0.264	(1.282)	1.672	(1.306)
Crimes reported per 100,000, county level, centered	- 0.004^∗∗^	(0.001)	- 0.003^∗∗^	(0.001)	- 0.004^∗∗^	(0.001)	- 0.004^∗∗^	(0.002)
Activity resource density by Crime interaction	0.000	(0.000)	0.001	(0.001)	0.001	(0.000)	0.001^∗∗^	(0.000)
Intercept	- 614.139^∗^	(278.733)	- 551.610^*‡*^	(282.031)	- 1076.852^∗∗^	(207.435)	- 880.393^∗∗^	(228.512)
*N*	1661		1661		1772		1772	
*R*^2^	0.464		0.423		0.425		0.376	
*F*	*F*_(15,113) _= 33.153		*F*_(13,115) _= 27.518		*F*_(15,112) _= 15.917		*F*_(13,114) _= 14.073	
**Hedonic regressions for 3**^***rd ***^**and 4 **^***th ***^**income quartiles**								
	**3**^***rd ***^**Quartile**				**4**^***th ***^**Quartile**			
**Variable**	**Coef**	**(Std. err.)**	**Coef**	**(Std. err.)**	**Coef**	**(Std. err.)**	**Coef**	**(Std. err.)**
Natural log of density, block group level	5.090^∗∗^	(1.549)	6.132^∗∗^	(1.899)	13.733^∗∗^	(4.578)	15.676^∗∗^	(4.870)
Proportion 25+ with a college degree	80.972^∗∗^	(17.629)	68.813^∗∗^	(19.332)	83.874^∗∗^	(23.607)	66.379^∗∗^	(24.693)
Per capita income, block group level	0.004^∗∗^	(0.001)	0.004^∗∗^	(0.001)	0.006^∗∗^	(0.001)	0.007^∗∗^	(0.001)
Median year housing built	0.359^∗^	(0.178)	0.181	(0.193)	-0.395	(0.353)	- 0.621^*‡*^	(0.333)
West (1=yes; 0=no)	39.481^∗∗^	(11.579)	32.112^∗^	(13.391)	64.383^∗^	(26.105)	63.959^∗^	(25.560)
Midwest (1=yes; 0=no)	-11.640	(9.834)	- 20.222^*‡*^	(11.103)	-8.557	(13.208)	-12.041	(13.671)
South (1=yes; 0=no)	- 19.936^∗^	(9.619)	- 26.150^∗^	(10.710)	- 28.036^*‡*^	(15.690)	- 31.458^∗^	(15.330)
Natural log of supermarket density	-3.867	(3.898)	0.129	(3.264)	9.647	(7.021)	14.727^∗^	(6.887)
Natural log of natural/health food store density	-1.893	(3.549)			1.370	(5.040)		
Natural log of fruit/vegetable market density	27.893^∗∗^	(4.130)			24.695^∗∗^	(6.120)		
Natural log of fast food (chain) density	- 5.820^∗∗^	(1.835)	- 7.437^∗∗^	(2.295)	- 13.022^∗∗^	(4.590)	- 15.682^∗∗^	(4.628)
Natural log of convenience store density	-2.628	(1.883)	1.105	(2.032)	- 10.322^∗^	(4.871)	-6.563	(4.236)
Natural log of activity resource density, centered	1.763	(2.041)	4.565^*‡*^	(2.367)	16.350^∗^	(6.526)	22.061^∗∗^	(6.921)
Crimes reported per 100,000, county level, centered	- 0.004^∗∗^	(0.001)	- 0.005^∗∗^	(0.001)	- 0.008^∗^	(0.003)	- 0.009^∗^	(0.003)
Activity resource density by Crime interaction	0.000	(0.001)	0.001	(0.001)	0.001	(0.002)	0.000	(0.002)
Intercept	- 703.002^*‡*^	(357.401)	-350.978	(386.007)	684.759	(687.906)	1120.822^*‡*^	(651.149)
*N*	1802		1802		1763		1763	
*R*^2^	0.419		0.357		0.520		0.507	
*F*	*F*_(15,111) _= 14.956		*F*_(13,113) _= 8.26		*F*_(15,109) _= 21.092		*F*_(13,111) _= 25.89	

Note: Numerator and denominator degrees of freedom for the *F* tests reflect, respectively, regression parameters minus 1 (*k*), and design degrees of freedom given the sampling frame, minus *k*. Significance levels: *‡* : 10% ∗ : 5% ∗∗ : 1%

People living in neighborhoods in the 1^*st*^ and 2^*nd*^ quartiles of income are willing to pay a small premium to avoid fast food outlets. Results from the first model for quartile two show that a 10 percent increase in access to fast food restaurants (density) is associated with housing values that are $358 lower ($479 in 2012 dollars).

Although people pay a premium to avoid crime in neighborhoods in the 1^*st*^ and 2^*nd*^ quartiles of income, they are not willing to pay a premium for access to PA resources. As noted earlier, this may be due to the fact that in low-income areas, parks are perceived as places for criminal activity [12].

The lower half of Table 3 shows the results for census blocks with incomes in the 3^*rd*^ and 4^*th*^ quartiles, revealing some differences in comparison to the estimates in the upper half of Table 3. Supermarket density is associated with housing price for the 4^*th*^ quartile of income when natural food and fruit and vegetable store densities are excluded. A 10 percent increase in supermarket density is associated with $1,473 ($1,966 in 2012 dollars). In the 3^*rd*^ quartile, a 10 percent increase in fruit and vegetable store density is associated with $2,789 higher housing costs ($3,727 in 2012 dollars).

The premium for avoiding convenience stores is inconsistent across neighborhoods by income quartile, but there is a premium to avoid fast food in all models. For instance, in the first specification of the 4^*th*^ quartile model, a 10 percent increase in access to fast food is associated with $1,302 in lower housing values ($1,740 in 2012 dollars).

Finally, wealthier neighborhoods also pay a higher premium for access to PA resources. In the full model for the 4th quartile, housing values are $1,635 higher ($2,184 in 2012 dollars) when activity resource density increases by 10 percent.

## Discussion

That the price of nearly identical dwellings in different neighborhoods can differ so dramatically reveals disparities in locational attributes. Our analysis reveals that physical attributes related to health are capitalized into higher housing values, *i.e.*, it costs more to live in those areas. Because the hedonic price model is essentially a bid model, capitalization can be thought of as evidence of preference, at the aggregate level, for a given neighborhood and its attributes versus alternative neighborhoods.

We found that access to fruit and vegetable stores are highly capitalized. For instance, a 10 percent rise in the density of fruit and vegetable stores, which could be one store opening, is associated with $2,907 higher home values ($3,884 in 2012 dollars). This result was consistent across neighborhoods with different income levels. Supermarket access was capitalized when variables for the densities of fruit and vegetable and natural food stores were excluded. However, fruit and vegetable store access always dominated supermarket access overall and across neighborhoods with different income levels.

In some cases, attributes which are detrimental to health are negatively capitalized, meaning housing values are discounted in those areas. In our study, people valued the avoidance of convenience stores and fast food chains. We found that a 10 percent rise in the density of fast food chains is associated with $625 lower home values ($884 in 2012 dollars).

We also found that capitalization varied by neighborhood income levels. Wealthier neighborhoods pay a higher premium for access to PA resources. Among neighborhoods in the 4th quartile, housing values are $1,635 higher ($2,184 in 2012 dollars) when activity resource density increases by 10 percent. Although people valued PA resources overall, activity resources are not consistently valued, especially among those with lower income. This implies that projects to improve access to parks and trails in a poor area may lead to wealthier people bidding up the value of nearby houses and apartments.

All neighborhood types paid a premium to avoid fast food, but the distaste premium increased by neighborhood income level. A 10 percent increase in access to fast food is associated with $1,302 ($1,740 in 2012 dollars) in lower housing values among neighborhoods in the 4^*th*^ income quartile. The distaste premium for convenience store access also increased by neighborhood income level.

Our results suggest that differential spatial access to health-related amenities is associated with capitalization. Capitalization has equity concerns in a bid model because wealthier persons will inevitably be able to outbid lower income people. Policies should encourage ubiquitous access to healthy food and PA opportunities, because without differential access, capitalization will not occur. Some policies exist to spread access, such as enterprise zones offering advantageous tax policies for supermarkets. Our results show that access to small fruit and vegetable markets are *more* valued than supermarkets, so subsidies to convenience stores, which themselves have negative valuation, to sell healthier foods would help equalize access. In terms of PA, smaller parks in several locations would be preferred to a single large park (or a few large parks). This is because access to a single large park is easier to capitalize.

While capitalization has equity concerns, our results offer a heretofore unavailed of method to reveal the value persons place on access to health-related amenities. The preference for access, particularly access for healthy food, extends to neighborhoods of all income levels.

Our hope is that in future research efforts, we will be able to use capitalization to control for selection, revealing the true health effects of the built environment.

## Limitations

One critical caveat to our findings is that they are relational, but not necessarily causal. For instance, an increase in the health-related amenities near a location can result in increased prices because the amenities make the location of the property more valuable. Even announced or prospective changes in an area’s amenities can affect housing pricing far before these additional amenities exist. For example, houses in a neighborhood may increase in value at the announcement of potential plans for building a neighborhood park, since this reflects the interest of developers in the area’s growth and improvement. Both are examples of increased amenities, actual or planned, driving housing prices.

However, directionality may also work in the opposite direction. For instance, in the scenario of owned condominiums or gated communities, the building of a new walking or exercise area may depend on whether there has been an increase in the buying value of these homes. The additional income generated by the upscaled prices may be part of a development plan for the condominium or gated community to improve the value of the group of homes, which will in turn, increase prices even further. Thus, it is likely that in some scenarios, amenities and prices affect each other reciprocally. Our data were from a single wave, but future research considering multiple waves of data on amenities and home prices may help distinguish directions of causality.

Additionally, our data reflect a particular historical period in the U.S. housing market, the early 2000s, which we chose because of the relative absence of bubbles and busts [32].

## Conclusions

In this study, we addressed a gap in the literature by linking research on the contextual factors that facilitate or impede a healthy lifestyle with the hedonic price framework, allowing us to estimate the degree to which these factors are capitalized into housing prices. Although the non-randomness of location is generally acknowledged in the health economics literature, our results highlight the extent and magnitude of the non-randomness. Our results show that, even among people in the lowest income quartile, access to better food comes at a premium. It is acknowledged that income and health are highly correlated, and our results show that the health benefits of better access to food among the poor comes at the cost of reduced income, net of housing costs. This perhaps explains the mixed results in the literature.

## Endnotes

^1^ Note, however, that there can also be high-income neighborhoods away from the city, since some prefer to build larger houses on several acres. However, buying the same house on the same number of acres, holding crime, schools, etc., constant, closer to the center of the city would cost more. For example, Victorian homes in Manhattan were eventually sold due to the increasing importance of New York City.

^2^ Note that the Tiebout model is more concerned with efficient taxation than capitalization [10].

## Competing interests

Both authors declare that they have no competing interests.

## Authors’ contributions

HSB conceived of the study and analytic methods, and participated in reviewing results. LMY carried out the analyses and conceived of adjustments to models. Both authors contributed to the writing of this manuscript, and approve the final manuscript.
